# Correlation analysis of land surface temperature and topographic elements in Hangzhou, China

**DOI:** 10.1038/s41598-020-67423-6

**Published:** 2020-06-26

**Authors:** Xiaoxue Peng, Wenyuan Wu, Yaoyao Zheng, Jingyi Sun, Tangao Hu, Pin Wang

**Affiliations:** 1 Institute of Remote Sensing and Earth Sciences, College of Science, Hangzhou Normal University, Hangzhou, 311121 China; 2 Zhejiang Provincial Key Laboratory of Urban Wetlands and Regional Change, Hangzhou Normal University, Hangzhou, 311121 China

**Keywords:** Climate-change ecology, Environmental sciences

## Abstract

In addition to human activities, this study found that topography is also an important factor affecting land surface temperature (LST). In this paper, based on Landsat 8 OLI/TIRS remote sensing images, a radiative transfer model was adopted to retrieve the LST, and a maximum likelihood method was used to remove artificial environmental interference factors, such as water bodies and built-up lands. This paper aims to analyze the influence of topographic factors, such as elevation, slope, aspect and shaded relief, on the LST of Hangzhou. By means of a statistical analysis, we obtained the quantitative relationship between these factors and constructed a multiple linear regression model of terrain factors and LST. The research revealed the following findings: (1) in the study area, elevation and slope are negatively correlated with LST, and all the factors have linear relationships with LST. (2) The relationship between aspect and LST is not significant, and high values of LST are found on the southern, southeastern and southwestern slopes; the lowest values are found on the northern slopes. (3) There is a significant linear relationship between the values of the shaded relief map and LST, and the more shadows there are, the lower the LST value will be. (4) After comprehensive analysis of the influence of the abovementioned topographic factors on the LST, it is found that shaded relief has the greatest contribution and is positively correlated with LST. The influence of shaded relief on surface thermal environment should be paid more attention in the process of surface thermal environment work. The assessment of the influence degree of shaded relief and surface thermal environment should be the premise and basis for many other studies.

## Introduction

Global warming has become one of the most important environmental problems faced by human beings. There is an urgent need to determine the influences of these factors and improve the surface environment on which human beings depend. At present, many directions have been opened in the study of the relationship between natural factors and land surface temperature (LST). For example, the relationship between vegetation and LST^1,2^, the relationship between vegetation, water and LST^3^, and the impact of urban land expansion and change on temperature^4^ are studied. In addition to the typical types of built-up lands, such as vegetation, water body and land for urban residents, the researchers found that topography may also be a factor affecting LST. For example, macroscopic geographical conditions (longitude, latitude, macroscopic climatic background conditions, etc.), local terrain conditions (elevation, slope, aspect, etc.) and the underlying surface properties (such as soil and vegetation status) can affect the surface temperature^5^. It has been concluded that there is a negative correlation between vegetation and land surface temperature (LST)^6^, and soil type is also closely related to the heating and cooling process of the land surface thermal environment^7^. Specifically, the influence of topography on the LST differs according to the amount of solar radiation received, and the effect of topography on the LST will have different influences over time^8^. With the development and popularity of studying LST, more attention was paid to the relationship between surface thermal environment and human, urban heat island, vegetation, fault zones and so on^9^. However, the relationship between LST and topography in regions was ignored. In fact, it is worth paying attention to how much this correlation affects and which factors in the topography have a decisive effect on the temperature. In the related research of surface thermal environment, thermal infrared remote sensing image was widely used. It can reflect true surface temperature field continuously, and it has the characteristics of low cost, wide coverage, rich data sources and so on. It is not limited by surface temperature observation conditions, and has many applications in geothermal resources exploration, volcano monitoring and other fields^10–13^.

Early studies focused on exploring the correlation between elevation and temperature and found that temperature tended to decline with increasing elevation^14^. Subsequently, a series of studies on slope and aspect further show that topography is an important factor in controlling LST. On a small scale, slope and aspect directly affect potential radiation and thermal load^15^. Mountain topography has a significant impact on LST. For example, in typical mountainous areas in southwestern China, LST is negatively correlated with elevation and slope, and the temperature on the southern slope is generally higher than that on the northern slope^16^. In addition, there is a quantitative relationship between topography and LST. For instance, LST can be expressed as a function of latitude, slope, aspect, terrain shadow and time^17^. On this basis, Sun established the optimal stepwise regression model of elevation, slope, aspect, solar incident angle, NDMI and LST^18^ and quantitatively analyzed the correlation between LST and topography for Mount Tai, providing a good reference for studying the spatial differentiation characteristics and formation mechanism of LST. In the research work, it is found that the elements in the terrain are more complex, and the correlation with LST and the evaluation of determining factors also need quantitative analysis. Some of the topographic factors do not affect the LST, but the terrain factors that really affect the LST and the degree of influence need to be discussed.

In view of this, with Hangzhou city as an example, this paper uses Landsat 8 OLI/TIRS remote sensing data, adopts a radiative transfer model to retrieve the LST, and uses a supervised classification method to remove artificial environmental interference to obtain LST information for the natural land surface. Based on Advanced Spaceborne Thermal Emission and Reflection Radiometer Global Digital Elevation Model (ASTER GDEM), terrain factors (elevation, slope, and aspect and a shaded relief map) are extracted and superimposed with LST data, and a quantitative model is used to describe the relationship between them. With the natural surface as the research object, this paper extensively analyzes the influence of topographic factors on the LST of Hangzhou city to provide a scientific basis for ecological environment construction and finding suitable living places for human beings. In order to show that the results do not have regional limitations and image limitations, we selected another scene Landsat 8 remote sensing image in Zhejiang Province, and carried out the same experiment to verify the relationship between topographic factors and temperature. Through the research work, the influence of topographic factors on the surface thermal environment is evaluated. Only in this way, in the process of all the surface thermal environment work, can we better evaluate the weight between the key terrain factors and other factors. This is also the premise and basis for other surface thermal environment work. It will bring some enlightenment to scholars' follow-up and more in-depth researches on such issues, and promote the emergence of more conclusions beneficial to human survival and development. In addition, the relevant research methods can be extended to the study of global change, which is of great significance for understanding the laws of nature and monitoring the surface thermal environment.

## Study area

**Figure 1:**
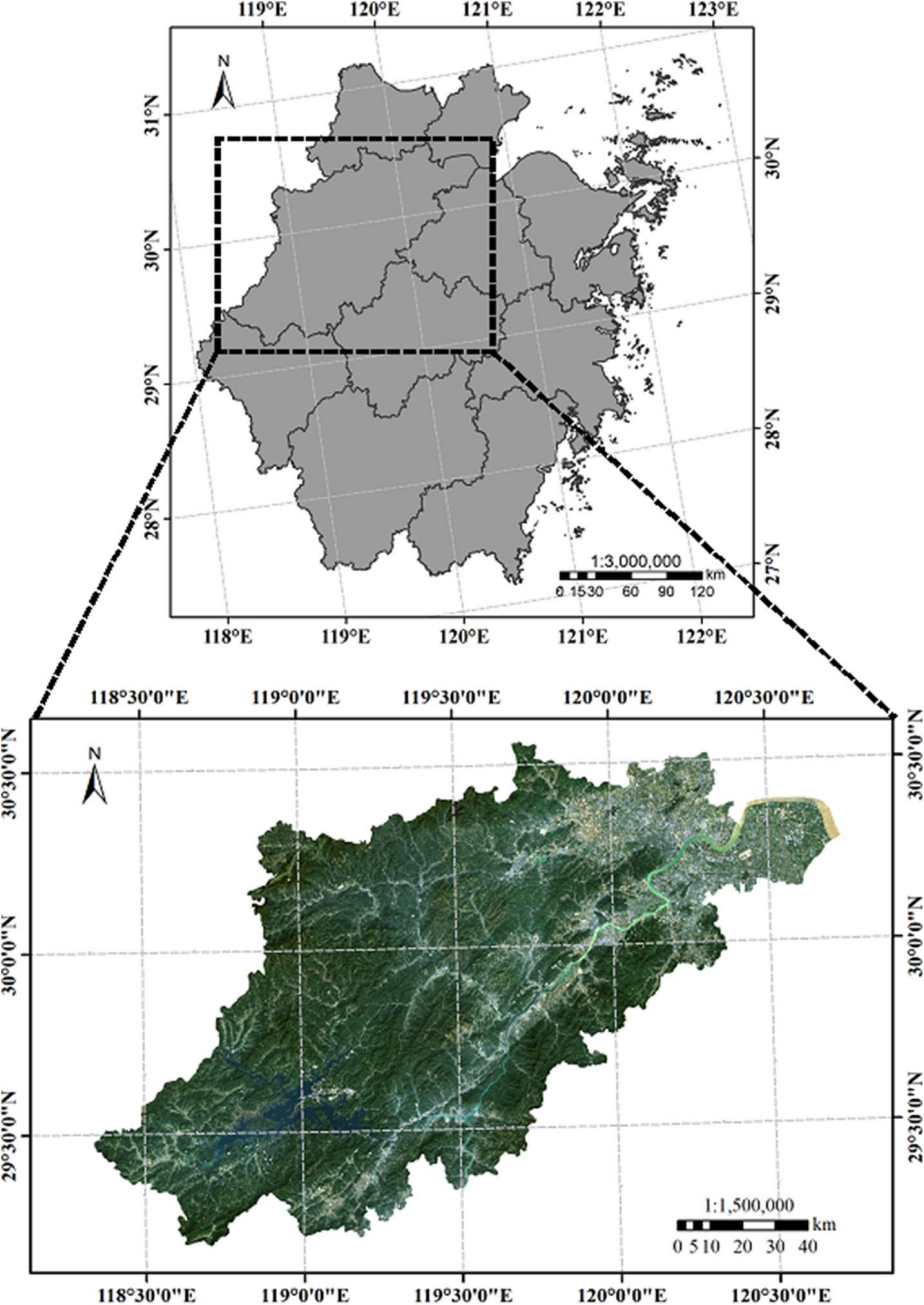
Location and topography of the study area. Map has been created with ESRI ARCMAP 10.0, available at https://support.esri.com/en/products/desktop/arcgis-desktop/arcmap/10.0.

Hangzhou, as one of the central cities in the Yangtze River Delta, is located in the north of Zhejiang Province and the lower reaches of the Qiantang River (Fig. 1). It is between 29°11′ N–30°33′ N and 118°21′ E–120°30′ E. The geographical coordinates of the downtown center are 30°16′ N and 120°12′ E. As of 2017, Hangzhou has ten districts (Shangcheng, Xiacheng, Jianggan, Gongshu, Xihu, Binjiang, Xiaoshan, Yuhang, Fuyang, and Linan), two counties (Tonglu and Chunan) and one county-level city (Jiande), with a total area of 16,853.57 km^2^. Hangzhou has the natural characteristics of typical cities in southern China in terms of topography and climate, and can be used as a representative city. Hangzhou has a subtropical monsoon climate. There are four distinct mild and humid seasons, among which spring and autumn last for a short periods and winter and summer are longer. According to a relevant survey, the annual average temperature of Hangzhou in 2016 was 18.2 °C, the average temperature in winter was 7.43 °C, the frost-free period was 230–260 days, and the annual precipitation was 1797.3 mm. In terms of topographic conditions, northwestern and southwestern Hangzhou include the Zhongshan hilly area in western Zhejiang Province, while northeastern and southeastern Hangzhou are part of the northern Zhejiang Plain, which has a dense river network and good topographic conditions. As a city surrounded by mountains on three sides, it is also an advantageous area suitable for the study of the relationship between topography and surface thermal environment. The periphery of the study area is mostly woodland and mountain areas with remarkable natural characteristics. And it is more helpful to study the influence of topography in natural factors on surface thermal environment. In addition, in the rapid development of Hangzhou, there is also a lot of research on the surface thermal environment. This makes the experiment better combined with all kinds of surface thermal environment research in the future, and promote the birth of more beneficial results.

## Materials and methods

### Research data

In this study, we use Landsat 8 OLI/TIRS thermal infrared (band 10) remote sensing data for LST inversion. Four Landsat 8 OLI/TIRS images covering Hangzhou with little or no cloud cover are selected. The imaging times include October 14 (path/row 120/39&120/40) and December 10 (path/row 119/39), 2013, and December 13 (path/row 119/40), 2014. Because three of the images only cover a few areas on the edge of Hangzhou. Therefore, the actual work is mainly based on the remote sensing image information obtained on December 10, 2013, which covers most of the area of Hangzhou (path/row 119/39). In order to verify whether the areas outside the study area will show the same regularity, the later experiment supplemented a scene of Landsat 8 data from other areas in Zhejiang Province to conduct a comparative experiment (path/row 118/40). The imaging time is December 3, 2013.Further, we used ASTER GDEM data to obtain terrain factors, such as slope, aspect and elevation, and a shaded relief map. ASTER GDEM data comes from the geospatial data cloud website (https://www.gscloud.cn). The research use Landsat 8 data with spatial resolution of 30 m in multi-spectral band and 100 m in thermal infrared band, which can match and correspond well with the spatial resolution of ASTER GDEM, so it is suitable to evaluate the influence of topography on surface thermal environment.

### Research methods

**Figure 2:**
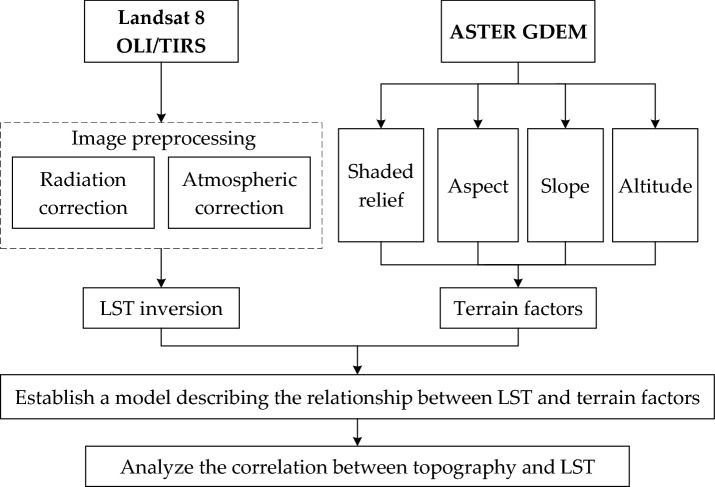
Technical research process.

We extracted the terrain factors, including the elevation, slope, and aspect and a shaded relief map, from the DEM data. Based on the Landsat 8 OLI/TIRS remote sensing data, the LST was retrieved. Combined with a supervised classification method to remove the artificial environmental interference factors, we obtained LST information for the natural environment of Hangzhou. Moreover, topographic factors, such as slope, aspect and elevation were superimposed and analyzed with the LST. A model describing the relationship between the LST and topographic factors was constructed by using statistics to evaluate the correlation between topography and LST in Hangzhou (Fig. 2).

### LST inversion

#### The radiative transfer model

In this paper, a radiative transfer model based on a single-channel algorithm^19,20^ is used to estimate the LST. The radiative transfer model is the basis of the whole LST inversion theory, which can be applied to the thermal infrared remote sensing data of any sensor^21^, reflecting the real LST, and has wide applicability^22–26^. Some studies also support this theory. They use radiative transfer equation method and universal single-channel algorithm to retrieve LST from Landsat 8 TIRS remote sensing data combined with meteorological data. The results showed that the inversion results of the radiative transfer equation method were closest to the measured values, while the universal single-channel algorithm was more suitable for the inversion of LST in the absence of real-time atmospheric profiles^27^. In addition, in order to further improve the accuracy of LST inversion, some scholars have improved the existing LST inversion methods and achieved some results. For example, for Landsat 8, a method of LST inversion based on Landsat 8 TIRS single-channel data is proposed, and the measured results show that the inversion accuracy of this method is high^28^. For the Landsat 8 OLI/TIRS remote sensing data used in the study, LST inversion by the radiative transfer model mainly includes the following processes: satellite data preprocessing (i.e., radiation calibration), TIRS band 10/11 radiance calculation, atmospheric correction, NDVI calculation, land surface emissivity calculation, blackbody radiance calculation (at the same temperature), and LST calculation^29^. The specific steps are as follows:

1 $${L}_{sensor,i}=\left[{\varepsilon }_{i}{B}_{i}\left({T}_{s}\right)+\left(1-{\varepsilon }_{i}\right){L}_{atm,i}\downarrow \right]{\tau }_{i}+{L}_{atm,i}\uparrow ,$$

where ${L}_{sensor,i}$ is the thermal infrared radiance of the ith band received by the sensor (W/(m^2^ sr μm)), ${\varepsilon }_{i}$ is the land surface specific emissivity of band i, ${B}_{i}\left({T}_{s}\right)$ is the Planck black body thermal radiance (W/(m^2^ sr μm)), ${T}_{s}$ is LST (K), ${\tau }_{i}$ is the total atmospheric transmittance from the ground to the sensor in band i, and ${L}_{atm,i}\downarrow$ and ${L}_{atm,i}\uparrow$ represent downward and upward radiation, respectively (W/(m^2^ sr μm)). The atmospheric parameters can be downloaded from the official website of NASA (https://atmcorr.gsfc.nasa.gov/), and ${\tau }_{i}$, ${L}_{atm,i}\downarrow$, and ${L}_{atm,i}\uparrow$ can be obtained from the imaging time, latitude and longitude corresponding to the image center.

According to Eq. (1), the blackbody thermal radiance ${B}_{i}\left({T}_{s}\right)$ can be obtained, and the final ${T}_{s}$ can be obtained by the following Planck formula:

2 $${T}_{s}=\frac{{K}_{2}}{\mathrm{ln}\left[\frac{{K}_{1}}{{B}_{i}\left({T}_{s}\right)}+1\right]},$$

where ${B}_{i}\left({T}_{s}\right)$ is the radiance value of a black body in the thermal infrared band. ${K}_{1}$ and ${K}_{2}$ can be obtained from the image header file (774.89 W/(m^2^ sr μm) and 1,321.08 K, respectively^30^).

One of the methods to obtain the surface emissivity is to divide the surface into different cover types in the remote sensing image, and then give different values to each surface cover type according to the measured or reported emissivity. In addition, the surface emissivity can also be estimated according to NDVI^31–33^. Due to the variety of surface features in the study area, a mixed image separation method is used to estimate the land surface emissivity^34^. In this method, the land surface is divided into three types: water, towns and natural surfaces. The emissivity of the water pixels is 0.995. The emissivity values of the towns and natural surfaces are obtained by the following equations:

3 $${\varepsilon }_{b}=0.9589+0.0860{P}_{v}-0.0671{{P}_{v}}^{2},$$

4 $${\varepsilon }_{s}=0.9625+0.0614{P}_{v}-0.0461{{P}_{v}}^{2},$$

where ${\varepsilon }_{b}$ and ${\varepsilon }_{s}$ represent the emissivity of urban pixels and natural surface pixels, respectively. ${P}_{v}$ is the vegetation coverage, which can be obtained by the following formula:

5 $$ P_{v} =  {\frac{{ {NDVI} - \left( {NDVI} \right)_{s} }}{{ {(NDVI)_{v} - \left( {NDVI} \right)_{s} } }}} $$

where NDVI is the normalized difference vegetation index and ${(NDVI)}_{v}$ and ${(NDVI)}_{s}$ are the NDVI values of vegetation and bare soil, respectively. Since the pixels covered by complete vegetation or bare soil in the study area are not obvious, $({NDVI)}_{v}$ and $({NDVI)}_{s}$ are set as 0.7 and 0.05, respectively, to approximate ${P}_{v}$. When the NDVI of a pixel is greater than 0.70, the value of ${P}_{v}$ is 1, and when the value is less than 0.05, the value of ${P}_{v}$ is 0; that is, when the value of NDVI in a pixel exceeds 0.70, the pixel is assumed to be completely covered with vegetation. When the value is less than 0.05, it means that the pixel is entirely bare soil.

Through these calculations, the LST data of the research area are obtained.

#### Interference removal

In order to better evaluate the impact of topographic factors on the surface thermal environment in the natural environment, we removed the interference of water bodies and built-up lands. In this paper, the maximum likelihood method is used to classify the objects in the study area. The maximum likelihood method is a widely used supervised classification method at present^4,35,36^. It classifies ground objects by calculating the mean values and covariance matrices of training sample data^37^. With the continuous development of land use classification methods of medium and high resolution remote sensing images, from the initial maximum likelihood method^4^ to the comprehensive classification method combined with auxiliary data^38–42^, and some new algorithms, such as decision tree, object-oriented, soft and hard classification^43–45^, can accurately obtain the land landscape pattern. This provides a certain technical basis for analyzing the influence of the change of surface types on the surface thermal environment. In this study, NDVI, MNDWI (Modified Normalized Difference Water Index) and NDBI (Normalized Difference Barren Index) are combined to classify images into five categories: built-up land, water, farmland, woodland, and bare land^46-48^. Moreover, the confusion matrix method is adopted to verify the classification accuracy with the help of high-resolution SPOT images. We used the Kappa coefficient to measure the accuracy of the classification. The kappa coefficient is a kind of index used for consistency checking and measuring classification accuracy, and its calculation is based on a confusion matrix^49,50^. The results show that the classification accuracy of the four images is more than 90%, and the Kappa coefficient is above 0.9, indicating a good classification. Using images from WRS-2 path/row 120/39 and 119/39 as examples, the values of the classification accuracy are 97.95% and 94.89%, and the Kappa coefficients are 0.96 and 0.91, respectively.

**Figure 3:**
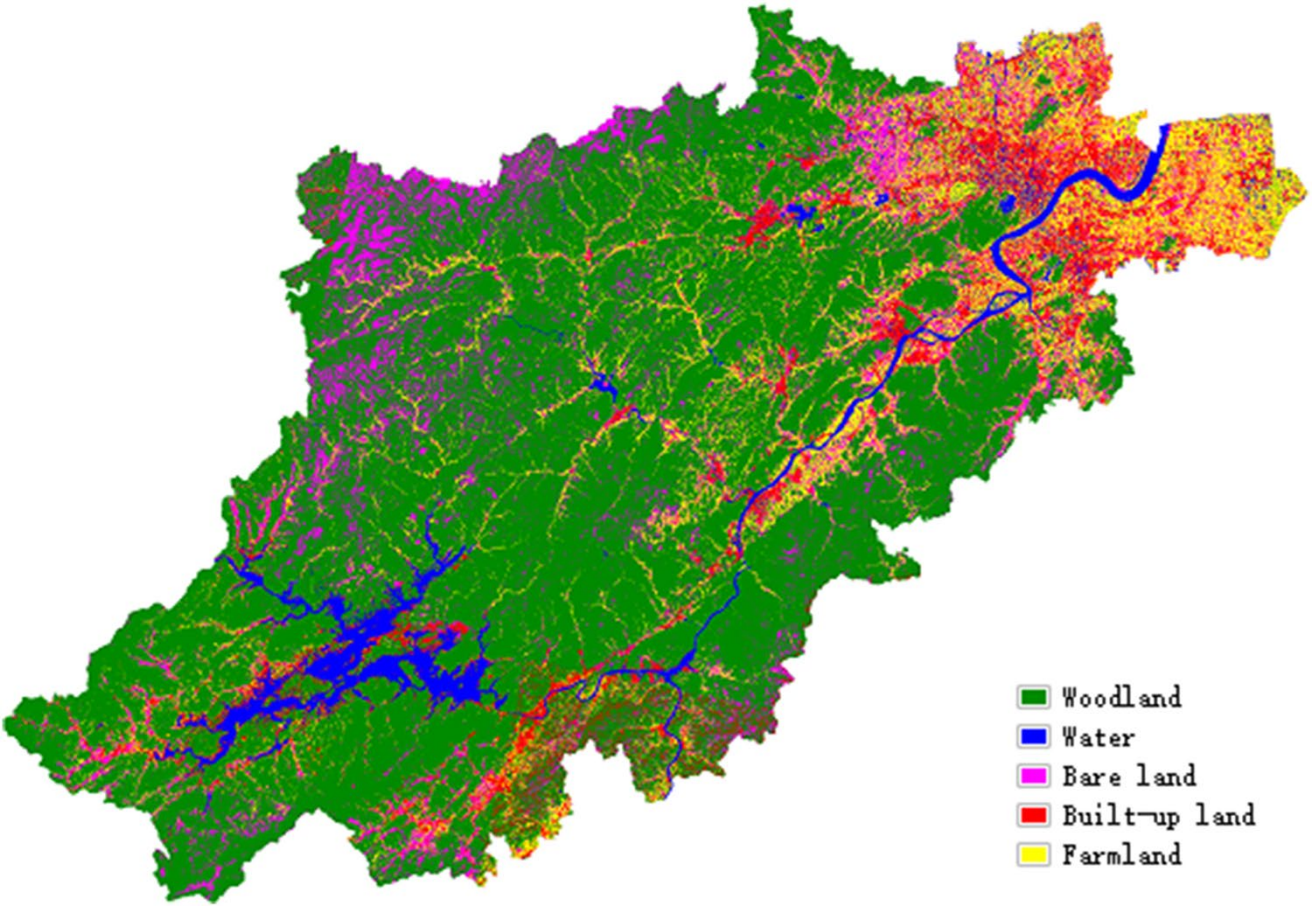
Classification results and accuracy verification of the maximum likelihood method. Map has been created with ENVI4.8/IDL8.0, available at https://www.harrisgeospatial.com/Software-Technology/ENVI.

After obtaining the ground object classification information (Fig. 3), the non-natural surface areas are extracted by using threshold extraction; that is, the built-up lands and water bodies are extracted from the classification results. They are then erased before the inversion of the LST to obtain the natural LST data of Hangzhou without interference from these factors.

#### Accuracy verification of the LST inversion results

**Figure 4:**
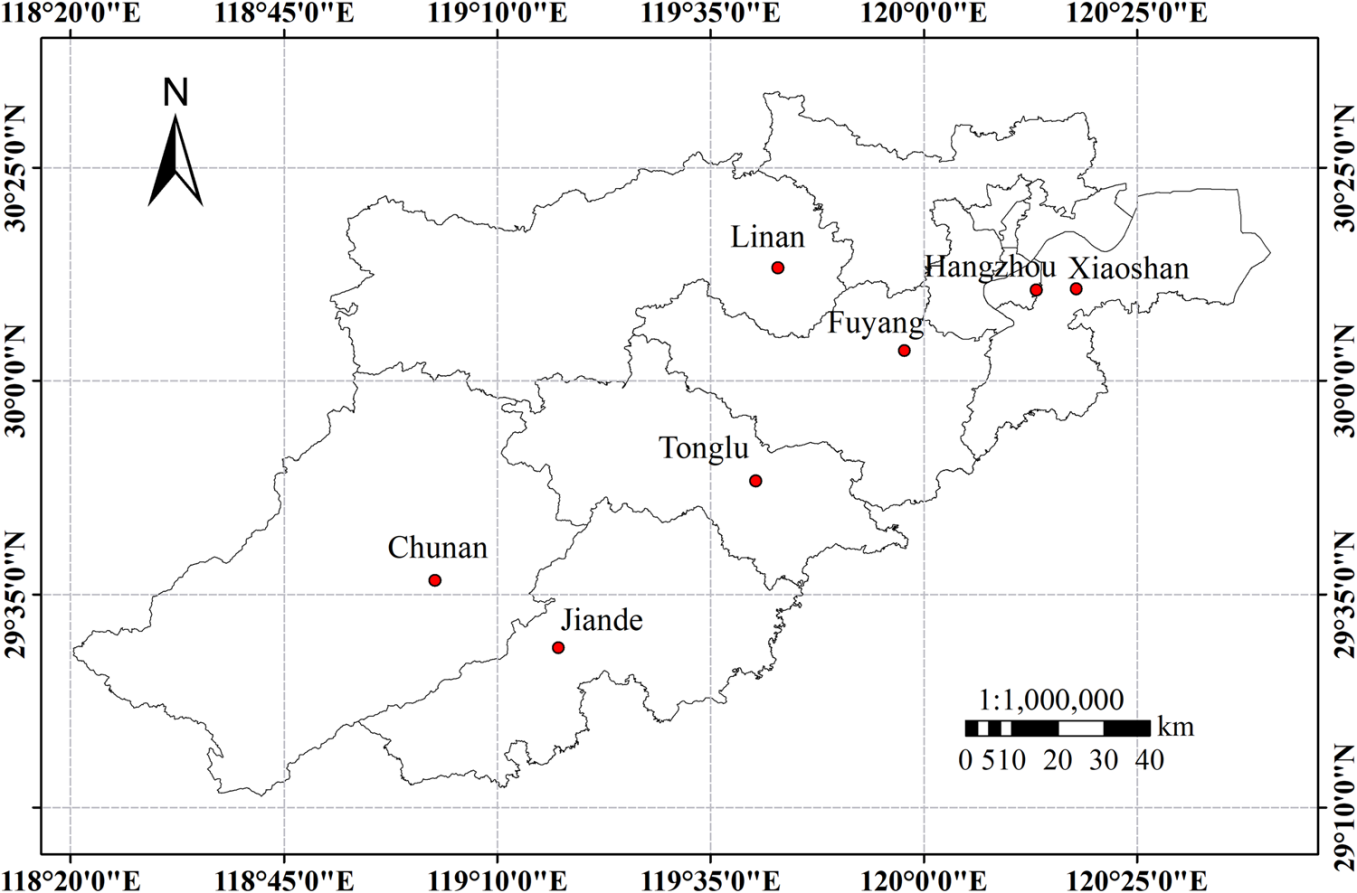
Distribution of the meteorological stations in the study area. Map has been created with ESRI ARCMAP 10.0, available at https://support.esri.com/en/products/desktop/arcgis-desktop/arcmap/10.0.

To verify the accuracy of the inversion, ground-based meteorological data corresponding to the estimated LST data from the Landsat 8 OLI/TIRS images are obtained through the China meteorological data network (https://data.cma.cn/). The meteorological data was measured at local time 10:00am on December 10, 2013. A total of seven verification points, including Linan, Fuyang, Hangzhou, Xiaoshan, Tonglu, Chunan and Jiande (Fig. 4), are selected to verify the accuracy of the LST inversion according to the measured meteorological stations. The specific measured temperature, estimated temperature and error information are shown in Table 1. The error between the LST inversion and the measured temperature is mostly less than 1 °C, indicating that the remote sensing-based LST inversion is not significantly different from the measured temperature overall. Therefore, this method, which is widely available, is suitable for subsequent correlation exploration and analysis.

**Table 1:** Accuracy verification of the Hangzhou LST inversion results.

Site ID	Meteorological stations	Location	The measured temperature (℃)	The inversion temperature (℃)	Error (℃)
58448	Linan	119.71°E, 30.22°N	6.04	5.06	0.98
58449	Fuyang	119.96°E, 30.06°N	6.13	6.55	− 0.42
58457	Hangzhou	120.22°E, 30.18°N	7.23	8.03	− 0.80
58459	Xiaoshan	120.30°E, 30.18°N	8.10	8.90	− 0.80
58542	Tonglu	119.67°E, 29.81°N	6.08	7.21	− 1.13
58543	Chunan	119.04°E, 29.61°N	6.67	6.11	0.56
58544	Jiande	119.29°E, 29.48°N	6.55	7.41	− 0.86

### LST and topography analysis

In the work, a set of numerical acquisition and superposition analysis method of LST and topography which is suitable for this study is obtained. For the inversion of the LST, we obtain the temperature data of the corresponding position of the grid layer in the study area by the way of vector distribution, and store the LST information quantitatively. According to the scope of the study area, use a rectangular grid with a specification of 500*500, place a vector point in each grid and give each vector point a fixed number. ASTER GDEM obtains quantitative data in the same way. And then remove all the rectangular grid points beyond the scope of the study area in order to better match with the area. A total of 14,377 valid data points were stored in the experiment. Finally, all the vector information points are derived as specific values, corresponding to LST and terrain information. And then import to the statistical analysis software for follow-up correlation analysis.

## Results

### The results of the superposition of the LST and terrain factors

**Figure 5:**
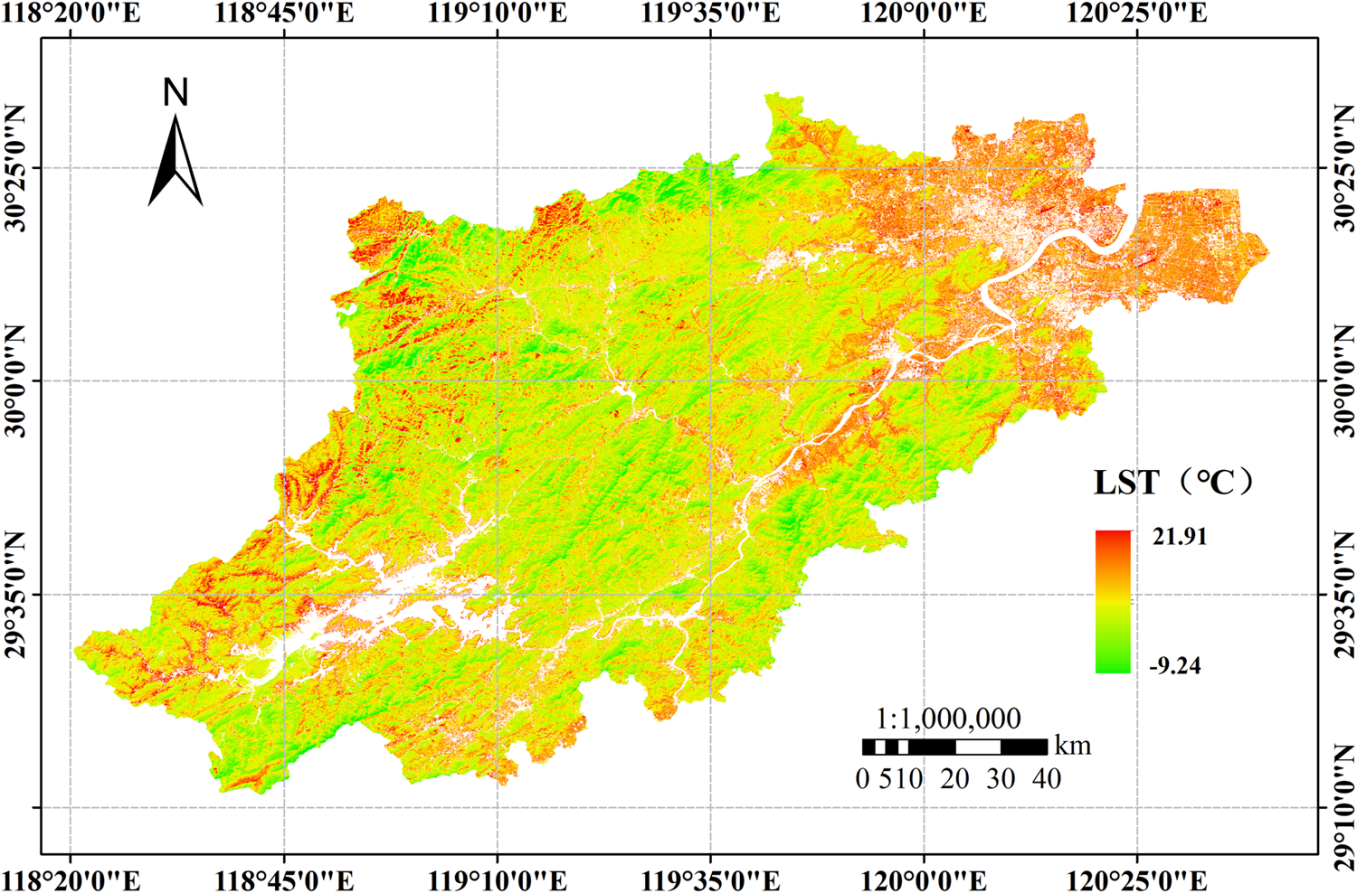
Natural LST data of the study area without interference factors. Map has been created with ESRI ARCMAP 10.0, available at https://support.esri.com/en/products/desktop/arcgis-desktop/arcmap/10.0.

The natural LST information of the study area was obtained by using a radiative transfer model combined with the maximum likelihood method (Fig. 5). As seen from Fig. 5, although the high-temperature areas of the built-up lands (associated with frequent human activities) and low-temperature water bodies are essentially removed, the high LST values in autumn and winter are still mainly focused around the built-up lands, and the LST shows a significant cooling trend in the natural land surface environment near the woodlands.

**Figure 6:**
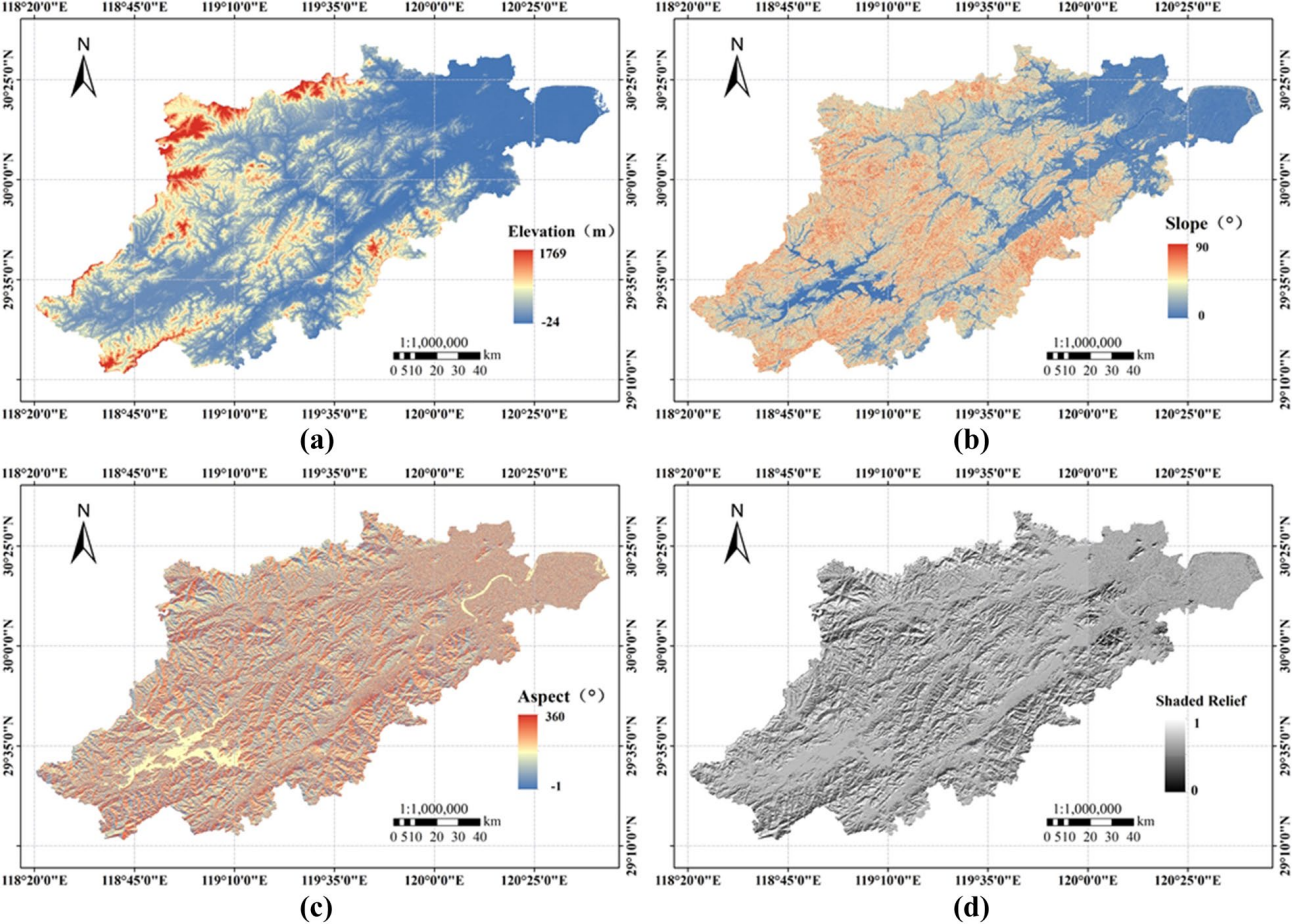
Terrain factor extraction results. (**a**)–(**d**) Extraction results for elevation, slope, aspect and the shaded relief map. Maps have been created with ESRI ARCMAP 10.0 + ENVI4.8/IDL8.0, available at https://support.esri.com/zh-cn/Products/Desktop/arcgis-desktop/arcmap/10 and https://www.harrisgeospatial.com/Software-Technology/ENVI.

Elevation, slope, and aspect and the shaded relief factor were extracted from the ASTER GDEM data (Fig. 6), the raster data (such as LST and terrain factor) were quantitatively processed, and the results were derived in numerical form to obtain the LST and terrain information corresponding to specific locations in the research area. A regression analysis was carried out by statistical methods, and the quantitative relationships between the variables were obtained by drawing a scatter diagram and data fitting.

### Single-factor analysis of the terrain effect on LST

#### Correlation between LST and elevation

**Figure 7:**
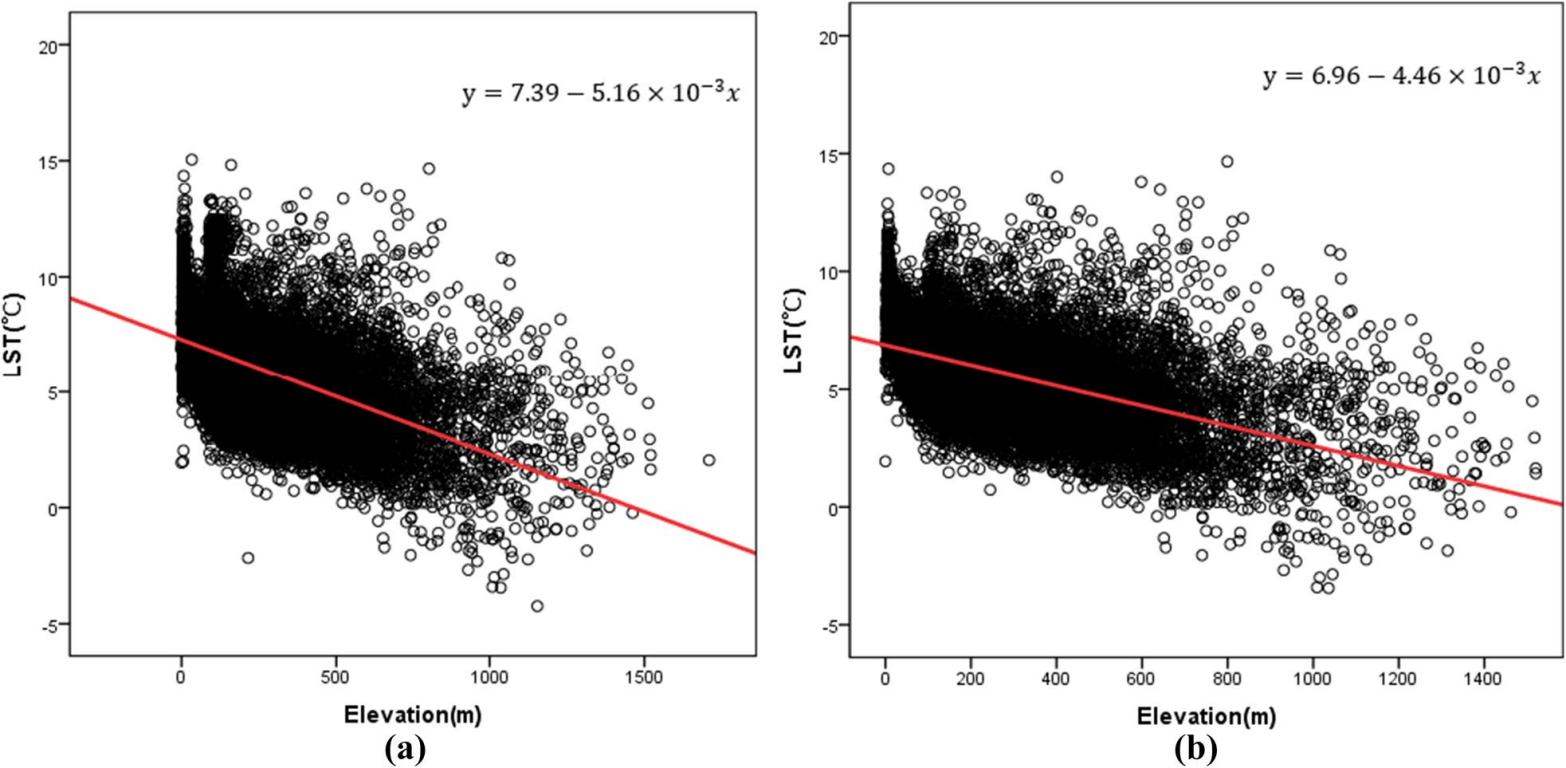
Regression analysis of the LST and elevation in the study area. (**a**) The results of the regression analysis between the LST and elevation. (**b**) The results of the regression analysis between the LST and elevation without interfering factors.

In this study, the correlation between LST and elevation data was discussed. A scatter diagram was drawn to obtain the results, as shown in Fig. 7. Figure 7a is the scatter diagram of the LST and elevation, and Fig. 7b is the scatter diagram of the LST and elevation excluding the artificial environmental disturbances, such as water bodies and built-up lands. The results show that there is a negative correlation between the LST and elevation, which is consistent with the results of existing research^51^.

Figure 7 shows that regardless of whether artificial environmental interference factors exist between the LST and elevation, there is an obvious trend that LST decreases gradually with increasing elevation. The regression analysis shows that the significance level in both cases is less than 0.01, and the linear regression relationship is obvious. The model correlation coefficient (R) in Fig. 7a is 0.551, and in Fig. 7b, it is 0.523. Based on this, when the effect of interference is removed, the LST of the study area decreases by approximately 5.16 °C for every 1 km rise in elevation. When interference is removed, the LST decreases by approximately 4.46 °C for every 1 km rise in elevation. That is, the comprehensive LST, including the artificial environment, declines faster with elevation than the LST in the natural environment, indicating that the comprehensive LST may change more significantly with elevation.

The main reasons for the influence of elevation on the LST are as follows. On the one hand, the influence of elevation on LST is mainly caused by air temperature, and the temperature value gradually decreases with increasing elevation. This pattern is observed mainly because the higher the troposphere atmosphere is from the ground, the less long-wave radiation energy it absorbs. The less heat the atmosphere stores, the lower the temperature is. However, the air temperature near the land surface shows a regular downward trend with increasing elevation, which in turn affects the heat exchange process between the surface and the air and finally acts on the surface heating processes^16^. On the other hand, considering the areas with higher elevations and larger topographic fluctuations, there are fewer human activities. That is, there are fewer anthropogenic disturbances, reducing the contribution of anthropogenic heat emissions to LST. Moreover, in this study area, the surface coverage at higher elevations is generally higher, and the cooling effect of green spaces makes the LST decrease with increasing elevation^1,52,53^.

#### Correlation between LST and slope

**Figure 8:**
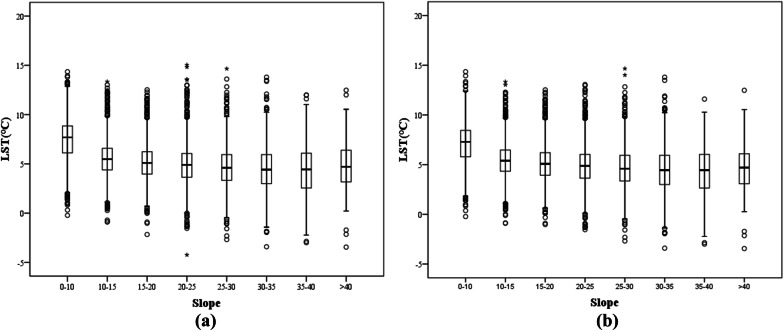
LST results of different slope ranges in the study area. The upper and lower black lines represent the maximum and minimum values, respectively. The third and fourth quantiles are represented by the upper and lower boundaries of the rectangle, respectively. The black line inside the rectangle represents the median, and the dots represent the outliers. (**a**) The statistical results of LST and slope, and (**b**) the statistical results of LST and slope without interfering factors.

In this study, the slope in the terrain model was divided into eight ranges: 0°–10°, 10°–15°, 15°–20°, 20°–25°, 25°–30°, 30°–35°, 35°–40° and > 40°. The charts of the slope and LST statistical analyses were created according to the corresponding slope grades (Fig. 8).

As shown in Fig. 8, LST generally follows a gradually declining trend with increasing slope. Figure 8a, b represent the statistically derived results of LST (including the artificial environment and natural LST without interfering factors, respectively) and slope. The median, minimum and third and fourth quantiles of the LST in each slope range mostly follow the abovementioned pattern; the maximum value deviates slightly but does not have a major impact on the overall trend.

**Figure 9:**
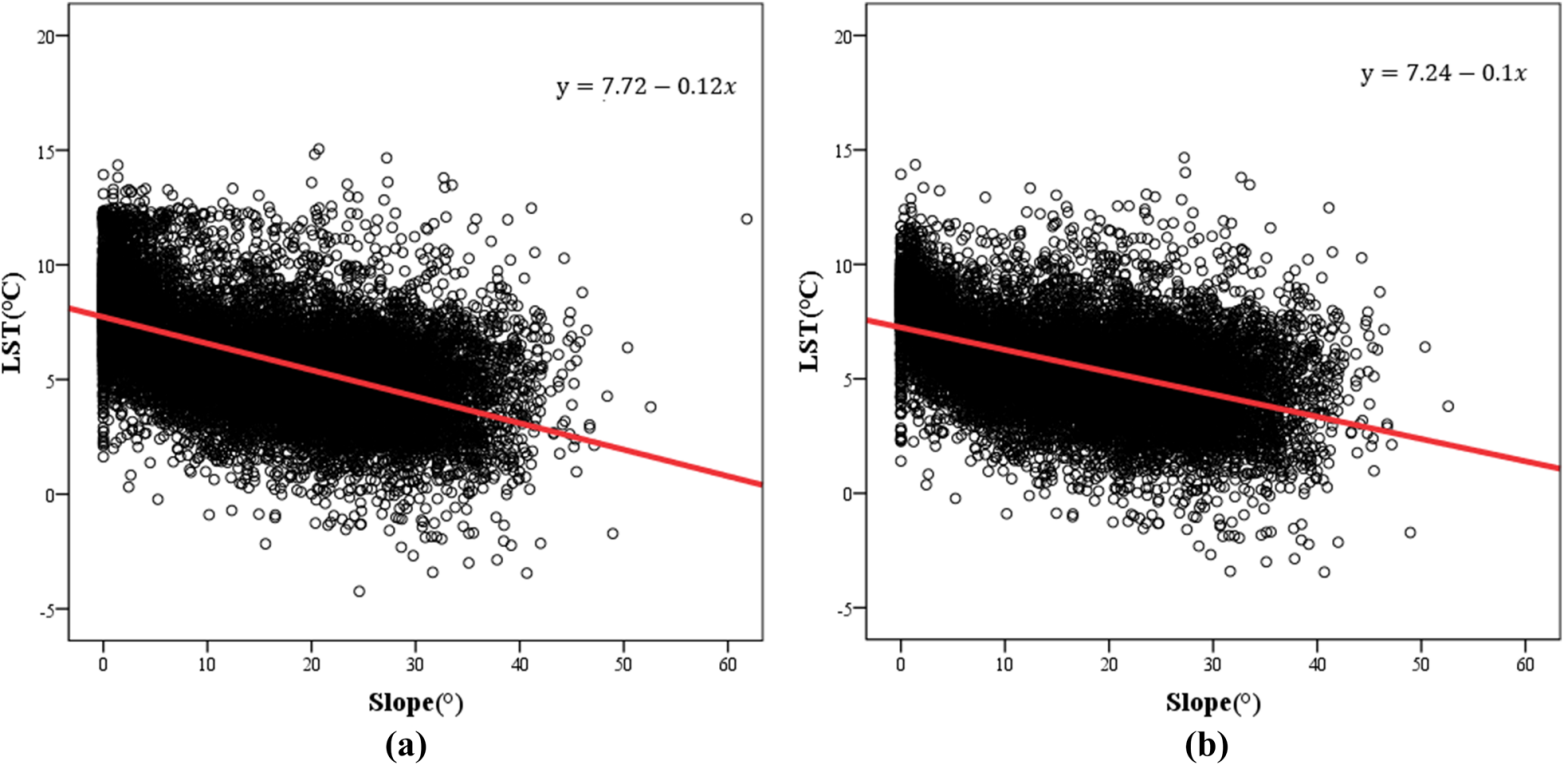
Regression analysis of LST and slope in the study area. (**a**) The results of the regression analysis between LST and slope. (**b**) The results of the regression analysis between LST and slope without interfering factors.

A scatter diagram of slope and LST was created (Fig. 9) to further analyze the quantitative relationship between LST and slope. The results of the linear regression analysis show that the significance level between the two variables is less than 0.01, indicating an obvious linear relationship. The model correlation coefficient (R) in Fig. 9a is 0.537, and in Fig. 9b, it is 0.483. The results show that when the effect of interference on the LST and slope is removed, the LST decreases by 0.6 °C for every 5° increase in slope. When the disturbance factors (i.e., water bodies and built-up lands) are removed from the study area, the LST decreases by 0.5 °C for every 5° increase in slope. Therefore, slope is related to LST, and the steeper the slope is, the lower the LST. Many studies have shown that different slopes affect the temperature of surface objects by influencing the incidence angle and reflectivity of solar radiation^54^. Of course, in the process of temperature inversion, the presence of slope will often affect the angular relationship between the sun, the surface and the sensor; each surface will be affected by the reflection from adjacent surfaces^55^, which is one of the reasons why slope affects the LST.

#### Correlation between LST and aspect

**Figure 10:**
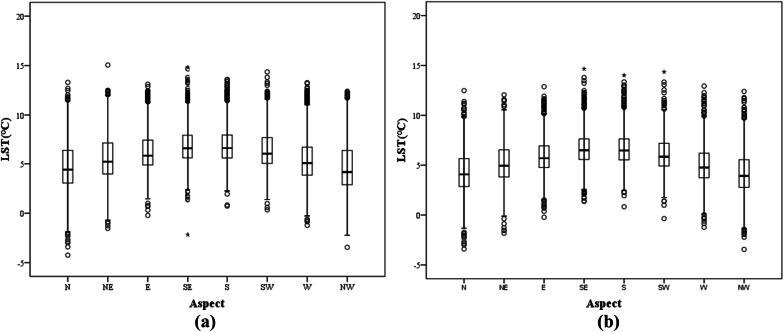
Statistical results of the analysis of the LST of different aspects in the research area. The upper and lower black lines represent the maximum and minimum values, respectively. The third and fourth quantiles are represented by the upper and lower boundaries of the rectangle, respectively. The black line inside the rectangle represents the median, and the dots represent the outliers. (**a**) The results of the statistical analysis between the LST and aspect, while (**b**) The results of the statistical analysis between the LST and aspect without the influence of the interfering factors.

In this study, the aspect was divided clockwise into the northern (N) slope, northeastern (NE) slope, eastern (E) slope, southeastern (SE) slope, southern (S) slope, southwestern (SW) slope, western (W) slope and northwestern (NW) slope. The correlation between aspect and LST was explored by drawing statistically based charts (Fig. 10). Figure 10a represents the comprehensive statistical results of the analysis between aspect and LST, including the influence of the artificial environment. Figure 10b shows the statistical results of the analysis between the natural LST and aspect, without the influence of interference factors. This analysis shows that there were high values of LST on the eastern slopes, southeastern slopes, southern slopes, southwestern slopes and western slopes, and peak values typically appear near southern slopes.

**Figure 11:**
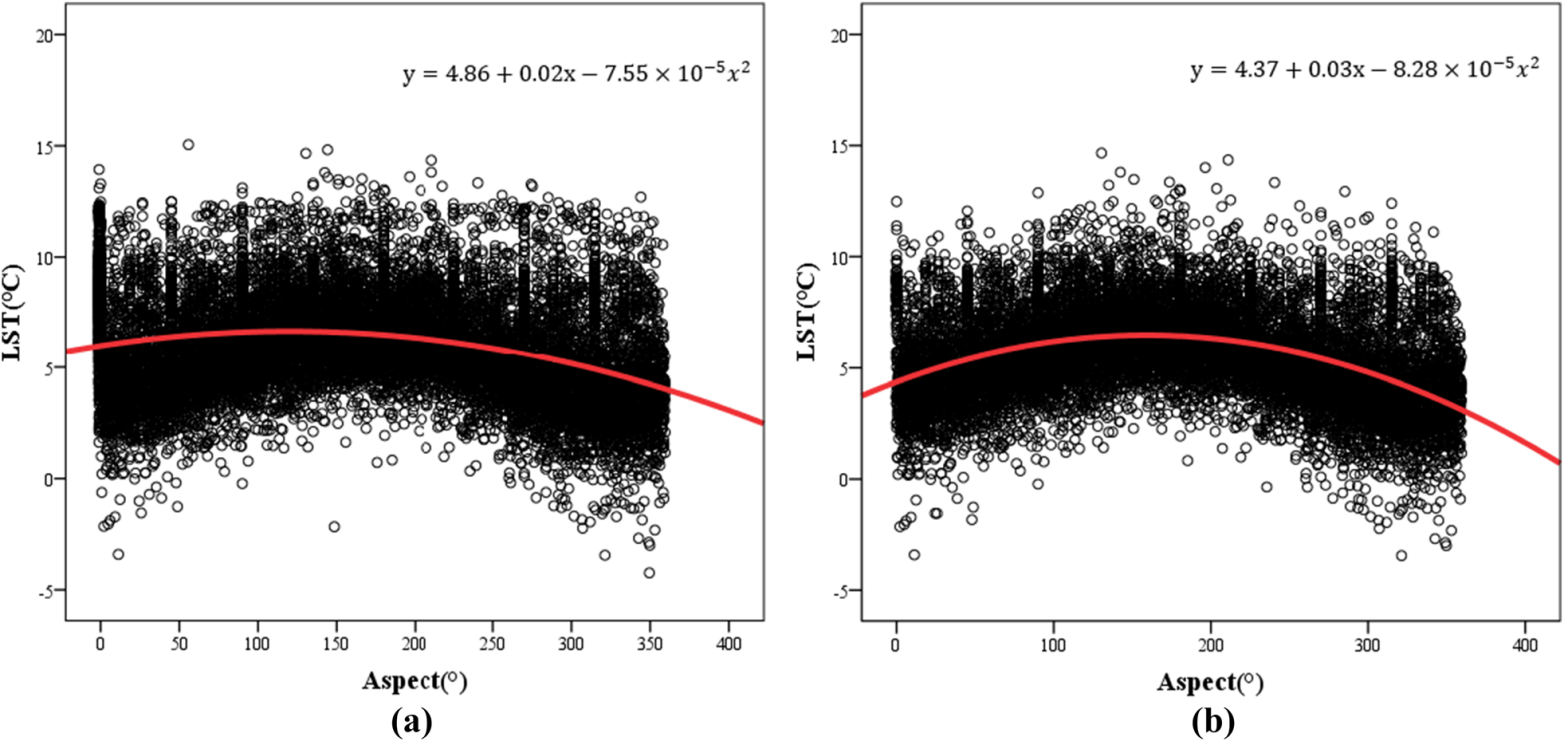
Regression analysis of LST and aspect in the study area. (**a**) The results of the regression analysis of LST and aspect. (**b**) The results of the regression analysis of LST and aspect without the influence of the interfering factors.

Further, a scatter diagram of aspect and LST was drawn, and the quantitative relationship between these factors was analyzed. The results are shown in Fig. 11. The regression analysis showed that in both cases, the significance of the result was less than 0.01, indicating that the model has significant meaning. The model correlation coefficient (R^2^) in Fig. 11a is 0.115, and in Fig. 11b, it is 0.162. The correlation is not obvious. However, the results do generally explain the trend of high temperatures on the southern slopes and low temperatures on northern slopes. This is consistent with existing conclusions that sunny slopes receive more direct solar radiation, resulting in high slope temperatures and intense evaporation^56^. In addition, northern slopes are mostly associated with shaded low-temperature areas in the terrain, which is also one of the reasons for the abovementioned trend.

#### Correlation between the LST and values of the shaded relief map

**Figure 12:**
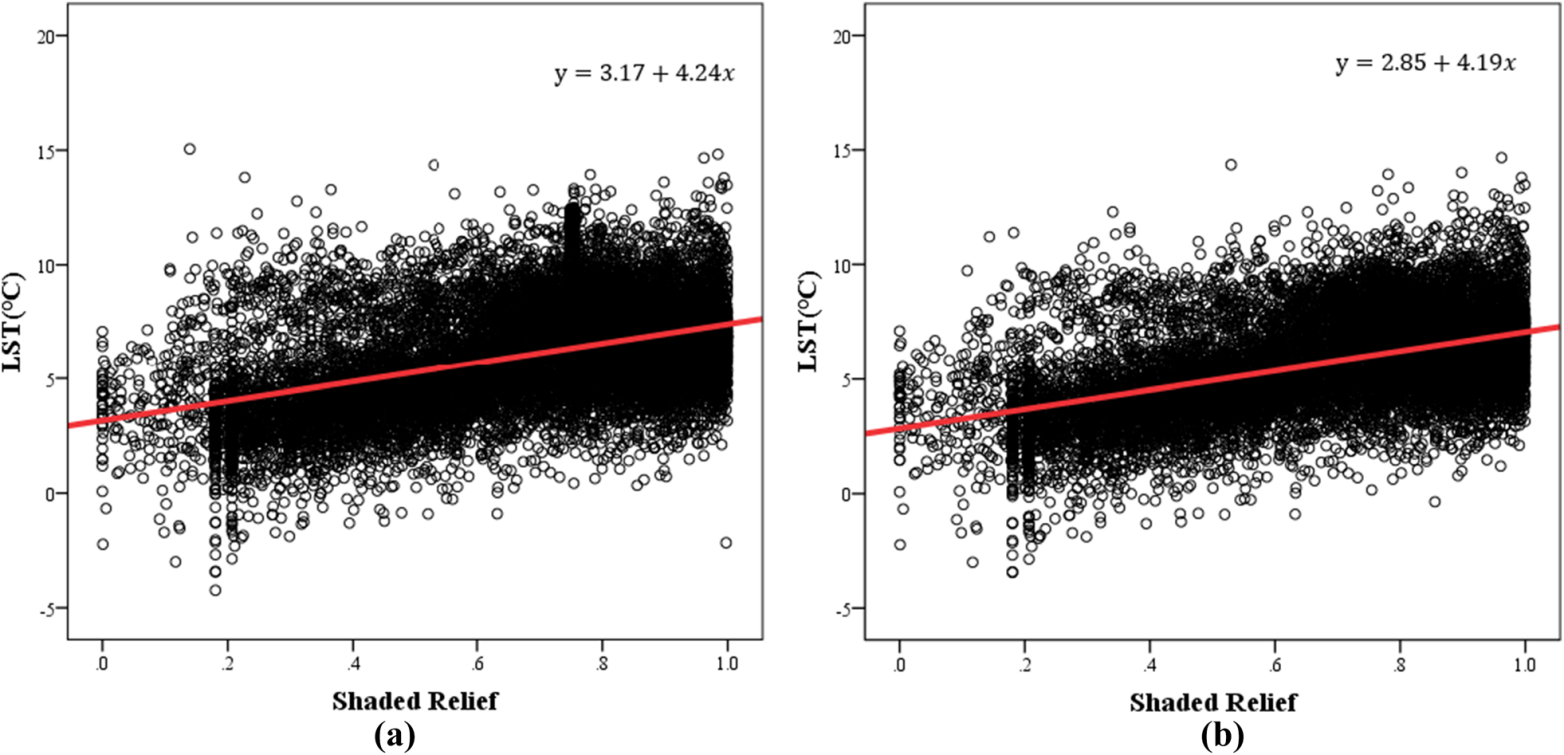
Regression analysis of the LST and values of the shaded relief map in the study area. (**a**) The results of the regression analysis of the LST and values of the shaded relief map. (**b**) The results of the regression analysis of the LST and values of the shaded relief map without the influence of the interfering factors.

Due to the influence of terrain fluctuation, when direct sunlight is partially or completely blocked and cannot reach a target, shadows will be formed in remote sensing images^57,58^. This work used the values of the shaded relief map to obtain information about the shaded geomorphology in the study area. Shaded relief is a cartographic technique that provides an apparent three-dimensional configuration of the terrain on maps and charts by use of graded shadows that would be cast by high ground if light were shining from the northwest. And it is the cosine of the sun incident angle and used in topographic correction method^59–63^. It can be calculated from the Topographic Model of the software ENVI. A value of 0 indicates that the corresponding pixel is almost completely covered in shadow^64^. A scatter diagram was drawn, and the correlation between the values of the shaded relief map and LST was quantitatively discussed with the addition of linear regression. The obtained results are shown in Fig. 12. Figure 12a represents the regression analysis results for the LST and values of the shaded relief map, and Fig. 12b represents the regression analysis results for the LST and shaded relief map without the influence of interfering factors. Studies have shown that the LST is positively related to the values of the shaded relief map, i.e., there is a negative correlation between the LST and shadows. Areas with greater coverage by shadows have lower LSTs. This is consistent with the previously reported trend that the shaded area has a lower LST due to the lack of sunlight and the accumulation of less solar radiation energy.

According to the regression analysis results, the model correlation coefficient (R) in Fig. 12a is 0.432, and in Fig. 12b, it is 0.482. The significance level of both is less than 0.01, so the results of the established model are significant. In addition, the removal of the disturbance factors has a certain influence on the quantitative relationship between the LST and values of the shaded relief map. By comparison, the LST changes more obviously with shadow topography under natural land surface conditions. It is believed that because the study area is in the Northern Hemisphere, the solar elevation angle in winter is smaller than that in summer, and the area in shadow on the remote sensing images is larger in the winter. In addition, compared with built-up lands, shadows in natural environments are mostly generated by rolling mountains, which leads to larger areas being covered by shadows, making the terrain effect more obvious.

### Diverse analysis of the terrain effect on the LST

The research on terrain factors includes elevation, slope, and aspect and shaded relief maps. Through the regression analysis of each terrain factor and the LST, it is found that the abovementioned topographic features are correlated with LST. They affect surface water and heat distribution patterns and remote sensing imaging processes by changing the path of light propagation^55,65,66^. However, in reality, the effect of topography on LST is usually not determined by a single topographic factor, and the regional difference in LST is often the result of the comprehensive influence of multiple topographic factors. Therefore, only by comprehensively considering the effects of topography, such as elevation, slope, aspect and shaded relief maps, can the actual effect of topography on the LST be effectively described.

Based on the results of the correlations between each topographic feature and the LST, a multiple regression analysis of the topographic factors and LST was conducted. A stepwise method was used to establish the related variables in the model, and the best fit model was the one in which all four types of terrain factors were considered. Therefore, LST is set as the observable variable (y), and it is assumed to be affected by nonrandom factors, such as elevation ($${x}_{1})$$, slope $${(x}_{2})$$ and aspect $$({x}_{3})$$ and the shaded relief values $${(x}_{4})$$. The unknown parameters are denoted as $${\beta }_{0}$$, $${\beta }_{1}$$, $${\beta }_{2}$$, $${\beta }_{3}$$ and $${\beta }_{4}$$, so the multiple linear regression model can be written as:

6 $$\mathrm{y}={\beta }_{0}+{\beta }_{1}{x}_{1}+{\beta }_{2}{x}_{2}+{\beta }_{3}{x}_{3}+{\beta }_{4}{x}_{4},$$

The unknown parameters were estimated by a least square method to minimize the error sum^67^. The significance of the regression equation and regression coefficient and the goodness of fit of the model were tested, and the results are shown in Table 2.

**Table 2:** Significance test of the linear regression.

R	R^2^	${R}_{\alpha }^{2}$	RMSE
0.706	0.498	0.498	1.529

As Table 3 shows, the significance levels of the regression equation and regression coefficient are less than 0.01, indicating that the linear relationship between the variables is obvious. The quality of the multiple linear regression model can be measured by the complex correlation coefficient (R), coefficient of determination (R^2^), corrected coefficient of determination (${R}_{\alpha }^{2}$) and root mean square error (RMSE). In this regression model, the complex correlation coefficient (R) is 0.706. These statistical results indicate that there is a close linear relationship between the independent variables and dependent variables^68^. The coefficient of determination and corrected coefficient of determination (R^2^) are both 0.498, indicating that the established model has a good fit. The RMSE is approximately 1.529, which reflects that the established model will have relatively high accuracy for predicting the values of the dependent variables. In conclusion, the results support the use of linear regressions to study the effects of diverse terrain on LSTs. Elevation, slope, aspect and the shaded relief values tend to have a comprehensive effect on LST. Finally, the multiple linear regression equation obtained from the nonstandardized coefficient is:

7 $$\mathrm{y}=4.996-0.003{x}_{1}-0.037{x}_{2}-0.001{x}_{3}+3.650{x}_{4},$$

where y is the LST (°C) and ${x}_{1}$–${x}_{4}$ represent elevation (m), slope (°), aspect (°) and the shaded relief value, respectively.

**Table 3:** Significance test of the regression coefficients.

Variable	Unstandardized coefficients		Standardized coefficients	t	Sig
	B	Std. error	Beta		
Constant	4.996	0.058		85.531	p < 0.001
Elevation (m)	− 0.003	0.000	− 0.381	− 49.605	p < 0.001
Slope (°)	− 0.037	0.002	− 0.182	− 23.427	p < 0.001
Aspect (°)	− 0.001	0.000	− 0.068	− 10.349	p < 0.001
Shaded relief	3.650	0.057	0.429	63.988	p < 0.001

According to the optimal regression equation, among the influencing factors of terrain on LST, the shaded relief value contributes significantly more than the other factors and is positively correlated with LST. This indicates that compared with elevation, slope and aspect, the shaded relief value plays a decisive role in affecting the LST in the study area, which cannot be ignored in similar subsequent studies.

Although Hangzhou is an important starting point for us to choose to show the relevant experimental results. However, in order to verify whether the above conclusions are only a special case of Hangzhou, we selected additional a remote sensing image in Zhejiang Province which are different from the location of the study area in this paper for supplementary experiments. The data are processed in the same way as the original experiment. After the regression analysis of shaded relief factor, it is found that the LST increases gradually with the increase of shaded relief value. The significance level was less than 0.01, and the correlation coefficient R was 0.593. This not only shows that the model established on this basis has good applicability, but also preliminarily shows that it plays a decisive role in the above topographic factors.

Then a comprehensive regression analysis was carried out between the above topographic factors and LST. This step-by-step method is used to establish the relevant variables of the model. When the four types of terrain factors are considered, the model fits best. Through the test of the significance of the regression equation and regression coefficient and the goodness of fit of the model, it is found that the coefficient of the independent variable slope forward in the regression model is 0.000. This may be because the slope direction accounts for very little in the comprehensive effect of topographic factors and does not play a major role in the changing trend of the overall surface temperature. Therefore, the diversified terrain effect of removing the slope component is further considered in the study, and the multivariate regression linear equation composed of the following non-standardized coefficients is obtained

8 $$\mathrm{y}=12.245+0.001{x}_{1}-0.032{x}_{2}+4.995{x}_{3},$$

In the formula, y is the LST (°C), and ${x}_{1}$–${x}_{3}$ represent the elevation (m), slope (°) and the shaded relief value, respectively. And the complex correlation coefficient R of the model is 0.616, which shows that the model has a certain practical application. However, no matter how the model changes, the, shaded relief factor plays a decisive role in the land surface temperature in the above topography. The supplementary experimental results further enhance the credibility of the original experimental results, indicating that the core results in this paper are not limited to Hangzhou area, its applicability in other area and images has been verified.

## Conclusions

In this paper, the influence of topography on LST is discussed. The natural LST was estimated based on elevation, slope, aspect and shaded relief values. The relationship between the variables was analyzed with single and multiple linear regressions, and a quantitative model describing the relationship between the variables was constructed. The following conclusions are drawn.

1. The LST of Hangzhou is obviously affected by elevation. In winter, the LST mainly decreases with increasing elevation. Moreover, there is an obvious linear relationship between these factors, and the comprehensive LST (i.e., including the artificial environment) changes more dramatically with increasing elevation than the natural LST.
2. The LST in Hangzhou is significantly affected by slope. Because different slopes affect the incidence angle and reflectivity of solar radiation, the LST mainly decreases with increases in slope in winter. The relationship between the two is linear, but whether the disturbance factors, such as water bodies and built-up lands, are removed has little influence on the quantitative relationship between slope and LST.
3. There is an obvious correlation between aspect and the LST in Hangzhou. The aspect influences the solar radiation received by a surface mainly by affecting the incidence angle of solar radiation and the length of time over which it reaches a surface. Overall, LST in the Northern Hemisphere follows a consistent trend: the highest temperature is found on the southern slopes, the second highest temperature is found on the southeastern and southwestern slopes, the third highest temperature is found on the eastern, western, northeastern and northwestern slopes, and the lowest temperature is found on the northern slopes. However, based on the regression analysis, the relationship between the aspect and surface temperature is not obvious.
4. There is also a significant correlation between the shaded relief values and LST in Hangzhou. The shaded areas, which have less accumulated solar radiation energy, have lower temperatures than the nonshaded areas. There is a significant linear relationship between these factors, and the larger the shaded area is, the lower the LST value.
5. Both the original experiment and the supplementary experiment show that compared with the influence of a single factor on the LST, the combination of elevation, slope, and aspect and the shaded relief values has a more significant, comprehensive effect on LST. Finally, a multiple linear regression model of terrain factors and LST can be constructed to quantitatively analyze the effect of terrain on the LST. In follow-up studies, the linear regression equations obtained by previous studies can be used to preliminarily predict the LST of a study area under known terrain conditions and obtain the distribution of the surface thermal environment under the influence of topography. In addition, among the abovementioned factors, the shaded relief map contributes more than the other variables and is positively correlated with the LST. It shows that the influence of shaded relief on surface thermal environment should be paid more attention in the process of surface thermal environment work. The assessment of the influence degree of shaded relief and surface thermal environment should be the premise and basis for many other studies.

All the above-mentioned studies show that there is a certain correlation between topography and LST. Therefore, to reach a more general conclusion, images from different periods and multiple scenes can be used in subsequent studies for in-depth discussions and analyses. Through the research work, the influence of topographic factors on the surface thermal environment is evaluated. According to the optimal regression equation, among the influencing factors of terrain on LST, the shaded relief value contributes significantly more than the other factors and is positively correlated with LST. This indicates that compared with elevation, slope and aspect, the shaded relief value plays a decisive role in affecting the LST in the study area, which cannot be ignored in similar subsequent studies. The influence of shaded relief on surface thermal environment should be paid more attention in the process of surface thermal environment work. The assessment of the influence degree of shaded relief and surface thermal environment should be the premise and basis for many other studies. We think in the future studies we will try to apply it to other remote sensing images and more area to evaluate the influence of topographic factors on the surface thermal environment. The research results can not only provide guidance for finding suitable places for human survival but also play a guiding role in the decision-making of relevant government departments, which is of great significance to the construction of ecological environments.
